# Site-Directed Spin Labeling EPR for Studying Membrane Proteins

**DOI:** 10.1155/2018/3248289

**Published:** 2018-01-23

**Authors:** Indra D. Sahu, Gary A. Lorigan

**Affiliations:** Department of Chemistry and Biochemistry, Miami University, Oxford, OH 45056, USA

## Abstract

Site-directed spin labeling (SDSL) in combination with electron paramagnetic resonance (EPR) spectroscopy is a rapidly expanding powerful biophysical technique to study the structural and dynamic properties of membrane proteins in a native environment. Membrane proteins are responsible for performing important functions in a wide variety of complicated biological systems that are responsible for the survival of living organisms. In this review, a brief introduction of the most popular SDSL EPR techniques and illustrations of recent applications for studying pertinent structural and dynamic properties on membrane proteins will be discussed.

## 1. Introduction

### 1.1. Site-Directed Spin Labeling EPR

Earlier biological EPR applications were limited to metalloproteins possessing paramagnetic centers or enzymes with radical cofactors. The absence of unpaired electrons in most biological materials would appear to minimize the application of EPR methods. Molecular biology techniques have been developed to incorporate stable radicals at specific locations on biological systems extending the application of EPR spectroscopy to nearly any biological system. These techniques are called spin labeling. The site-specific introduction of unpaired electrons into biomolecules in the form of spin labels is known as site-directed spin labeling (SDSL) [1, 2]. In SDSL experiments, all native nondisulfide bonded cysteines are removed by replacing them with another amino acid such as an alanine or serine. A unique cysteine residue is then introduced into a recombinant protein using site-directed mutagenesis and subsequently reacted with a sulfhydryl-specific nitroxide reagent to generate a stable spin label side-chain [2–4].

**Figure 1:**
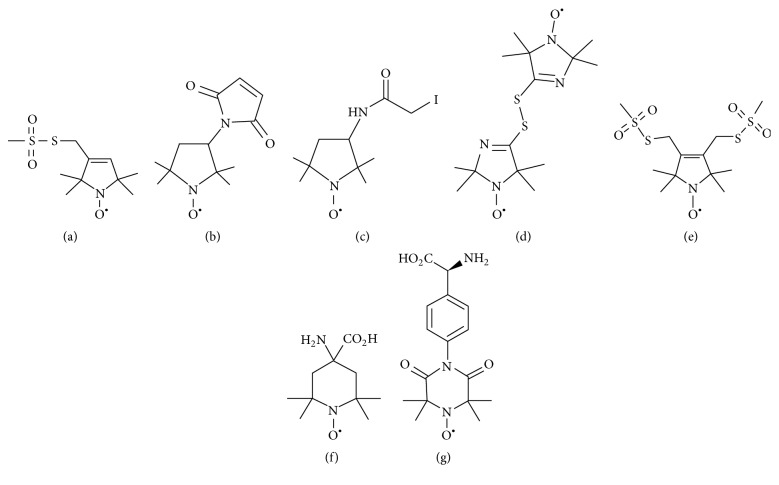
Structure of nitroxide spin labels used in the SDSL EPR study of micromolecules. (a) Methanethiosulfonate spin label (MTSL), (b) maleimide spin label (MSL) N-(1-oxyl-2,2,6,6-tetramethyl-4-piperidinyl) maleimide, (c) iodoacetamide spin label (ISL), (d) bis(1-oxyl-2,2,5,5-tetramethyl-3-imidazolin-4-yl) disulfide (IDSL), (e) bifunctional spin label (BSL), (f) 2,2,6,6-tetramethyl-N-oxyl-4-amino-4-carboxylic acid (TOAC), and (g) 4-(3,3,5,5-tetramethyl-2,6-dioxo-4-oxylpiperazin-1-yl)-l-phenylglycine (TOPP).

**Figure 2:**
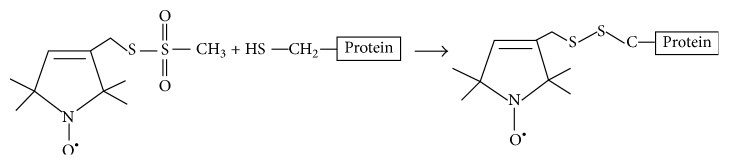
Structure of MTSL (methanethiosulfonate spin label) and the resulting side-chain produced by reaction with a cysteine residue on a protein.

Figure 1 shows the chemical structure of several nitroxide based spin label probes used for EPR spectroscopic studies of biomolecules [5–15]. The spin label probes in Figures 1(a)–1(e) are incorporated using site-directed mutagenesis while spin probes in Figures 1(f)-1(g) are incorporated using solid phase peptide synthesis. A resulting side-chain produced by reaction of the most commonly used spin label, methanethiosulfonate spin label (MTSL), with the cysteine residue of the protein is shown in Figure 2 [8]. We refer a recent book chapter authored by Haugland et al. for details of nitroxide spin labels used for SDSL EPR spectroscopy [6].

### 1.2. Origin of Spin Label EPR Spectrum

**Figure 3:**
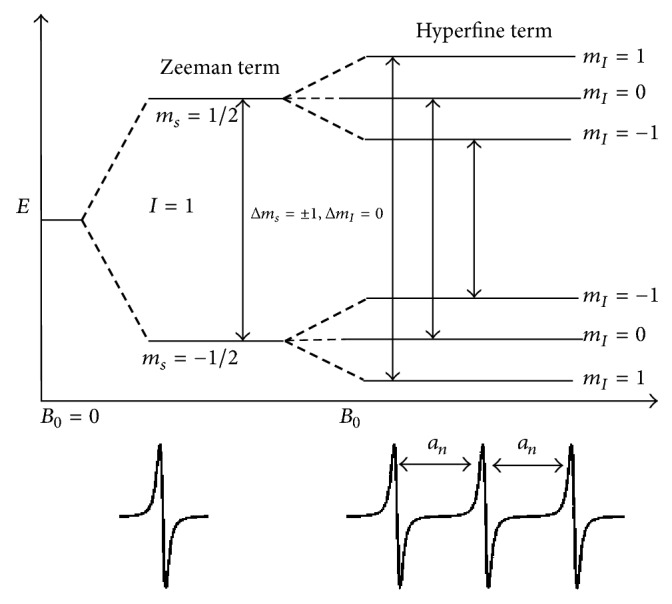
Energy level diagram of a nitroxide spin label in the presence of a magnetic field *B*_0_. The lower panel shows the corresponding EPR spectra in the absence and presence of a ^14^N (*I* = 1) hyperfine interaction.

EPR spectroscopy measures the absorption of microwave radiation corresponding to the energy splitting of an unpaired electron when it is placed in a strong magnetic field. Therefore, an EPR active sample requires the presence of an unpaired electron spin. The simplest EPR active system consists of a single unpaired electron spin residing in a molecular orbital. The electron possesses a magnetic moment and spin quantum number *S* = 1/2, with magnetic spin components *M*_*s*_ = +1/2 and *M*_*s*_ = −1/2. In the absence of a static magnetic field, these two states are degenerated and have the same energy. However, when an external magnetic field (*B*_0_) is applied, the magnetic moment of electron aligns itself either parallel (*m*_*s*_ = −1/2) or antiparallel (*m*_*s*_ = +1/2) to the field having a specific energy to each alignment. The parallel alignment corresponds to the lower energy state and the antiparallel alignment corresponds to the higher energy state as shown in Figure 3. The energy separation between the lower and the upper state is given by

(1) $$\begin{array}{c} \Delta E=g_{e}\beta B_{0}, \end{array}$$

where *g*_*e*_ is the electron's so-called *g*-factor which varies depending on the electronic configuration of the radical or ion that is similar to a chemical shift parameter in NMR and *β* is the electron Bohr magneton. The above equation (1) implies that splitting of energy levels is proportional to the magnetic field *B*_0_ strength as shown in Figure 3. An unpaired electron spin can flip between the two energy levels by absorbing microwave radiation of energy *hν*, obeying the fundamental equation of EPR spectroscopy [16]:

(2) $$\begin{array}{c} h\nu =g_{e}\beta B_{0}, \end{array}$$

where *h* is Planck's constant and *ν* is the frequency of the microwave radiation.

In a typical continuous wave- (CW-) EPR experiment, a fixed microwave frequency is applied and the magnetic field *B*_0_ is varied. An EPR transition occurs when the energy separation between the two electron spin states matches the constant microwave frequency (2). This phenomenon is known as resonance [16]. In addition to varying *B*_0_, the field is also modulated to improve the signal to noise of the spectra. This gives rise to the derivative lineshape typically observed in most EPR spectra. The EPR derivative spectrum is shown in the lower panel of Figure 3. The magnetic field at which this signal appears depends on the *g*-value, which dictates the slope at which the energy levels for the two spin states change as a function of the magnetic field. In most biological systems, the *g*-value is anisotropic with an orientation dependence meaning that the effective *g*-value is different depending on the orientation of the molecule with respect to the applied magnetic field.

If the free electron does not interact with the nearby nuclei, only one line is observed in the EPR spectrum (lower left panel in Figure 3). But for nitroxide spin labels used in most biological studies, the unpaired electron interacts primarily with the nitrogen nucleus (^14^N, nuclear spin (*I*) = 1). This interaction is called the hyperfine interaction (*A*) and depends on the amount of electron spin density on the nucleus, the distance between the electron spin and the nucleus, and the angle between the two with respect to the magnetic field (*B*_0_). This hyperfine interaction produces a small change in the allowed energy levels of the electrons and splits the EPR lines into multiple lines depending on the nuclear spin state. The hyperfine splitting due to coupling of an electron to a single ^14^N nucleus (*I* = 1) is shown in Figure 3. Other parameters that can influence an EPR spectrum are electron-electron couplings between two sets of spins that can provide valuable distance information in biological systems.

EPR spectroscopy is a very sensitive technique providing up to three orders of magnitude higher sensitivity when compared to nuclear magnetic resonance (NMR) spectroscopy. It can be applied to any size protein without relying on expensive isotopic labels [17]. It is not influenced by the optical properties of the sample. EPR experiments can be performed on a wide range of samples from proteins in solution to highly packed membrane suspensions, tissue samples, ammonium sulfate-precipitated solids, or samples frozen and maintained at cryogenic temperatures [18]. EPR experiments can be conducted at low volume and concentration (~70 nanoliter to several mL sample or even small animals [19, 20]). EPR spectroscopy can answer pertinent structural and dynamic questions related to both solution and membrane bound proteins that are very challenging to be obtained by traditional biophysical methods [21–24]. CW-EPR spectroscopy of spin labeled molecules reveals structural and dynamic information about the motion of the nitroxide side-chain, solvent accessibility, solvent polarity, and intra- or intermolecular distances between two nitroxides or a single nitroxide and another paramagnetic center in the system [3, 8, 15, 22, 25]. The lineshape analysis of the EPR data for a series of spin labeled protein sequences can probe the structural properties of the protein at backbone level spatial resolution [26–29].

### 1.3. Membrane Proteins

Membrane proteins are responsible for the exchange of signals and physical materials across the membrane and play an essential role in different aspects of cellular activities [30, 31]. Membrane proteins comprise 30% of sequenced genes [32–34]. Mutations in genes and misfolding of membrane proteins are associated with numerous human dysfunctions, disorders, and diseases [35, 36]. Approximately, half of all the FDA approved drugs target membrane proteins [37, 38]. Detailed structural and dynamic information for membrane proteins are vital for understanding intermolecular interactions, protein functions, and regulation [8, 22, 39]. Despite the abundance and clear importance of membrane proteins, very limited knowledge about these systems exists [40, 41]. Membrane proteins can interact with a lipid bilayer in various different fashions or orientations to maintain functional stability. The membrane interacting protein helices may be of varied length or curved in the middle of the membrane bilayer. They may lie flat on membrane surface, cross the membrane at different angles, or form reentrant loops.

X-ray crystallography and nuclear magnetic resonance (NMR) spectroscopy are the two most successful and popular biophysical techniques used to probe structural information on protein systems. NMR spectroscopy is also used to obtain dynamic information for a variety of biological systems. Solution NMR can be used to probe the protein structure in a physiologically relevant condition; however, a larger size protein (>~50 kD) is difficult to study using this technique [5, 42–44]. NMR structural studies on membrane proteins are also difficult due to the size of the micelle complex and higher spectral linewidth [45]. X-ray crystallography provides highly resolved structural information but cannot provide detailed dynamic information [46]. In addition, the hydrophobic nature of membrane protein often complicates the process of crystallization, introducing challenges for X-ray crystallographic techniques for studying many membrane proteins [43]. EPR spectroscopy is a rapidly expanding and powerful biophysical technique to resolve these challenges and provides prominent solutions to glean structural and dynamic information on peptides, proteins, macromolecules, and nucleic acids [3, 8, 12, 17, 21–23, 47–50]. 

## 2. Application of SDSL EPR Techniques for Studying Membrane Proteins

SDSL in combination with EPR spectroscopy has been widely used to study membrane proteins. This is a very wide topic, which will be discussed in an introductory fashion with recent illustrations in the following sections. For more in-depth information, the following are excellent reviews [8, 12, 15, 22, 24, 54].

### 2.1. Membrane Protein Dynamics and Topology

The flexible nature of the MTSL nitroxide spin label provides its reorientation motion highly dependent on neighboring side-chains and secondary structure components in its immediate environment and hence can report local structure of the protein. The lineshape of the room temperature EPR spectra reflects the mobility of the spin label side-chain and its relation to protein structures. Spin labeled sites exposed to bulk water show reorientational correlation times of spin label side-chains resulting in very sharp EPR spectral peaks with small linewidths of the central lines. On the contrary, a spin label with very slow motion will be in the rigid limit of the spectrum [55]. In the rigid limit, sample is frozen and the full orientation-dependent parameters are observed. For systems in which the spin label movement falls between these two regions, dynamic properties of the spin label located at the specific site can be described in the terms of a correlation time (*τ*_*c*_) [55]. The overall mobility of the nitroxide spin label attached to the protein or peptide is a superposition of the contributions from the motion of the label relative to the peptide backbone, fluctuations of the *α*-carbon backbone, and the rotational motion of the entire protein or peptide. Under experimental conditions, these motions can be isolated from the EPR spectrum. The inverse linewidth of the central line of the EPR spectrum provides a measure of relative mobility [22, 25, 51, 56]. A plot of the inverse linewidth mobility against the amino acid sequence can produce a periodic data profile, which can be used to predict the local secondary structure of the proteins and peptides [8, 22, 51, 57].

The changes in the spin label mobility can be used to investigate the peptide binding to the membrane [54, 58]. In the solvent phase, a spin labeled peptide or small protein rapidly tumbling leads to an isotropic spectrum with a rotational correlation time of less than nanosecond. However, in a membrane, spin labeled peptides experience restricted mobility, resulting in a broader EPR spectrum with two motional components resulting from the superposition of the signals arising from a free and bound peptide [51, 54, 59–61]. Protein topology in a membrane can be studied with respect to the membrane using nitroxide based SDSL EPR power saturation experiments [8, 60–62]. This method can also be used to identify functional domains in membrane proteins [46]. There are several biologically important membrane proteins such as the prokaryotic potassium channel KcsA, KCNE1, lactose permease protein, integrin *β*_1a_, C99 domain of the amyloid precursor protein, bacteriorhodopsin, and KvAP voltage-sensing domain that have been studied in a membrane environment using nitroxide based SDSL CW-EPR spectroscopy to probe the structural topology and dynamic properties [51, 56, 61, 63–67].

**Figure 4:**
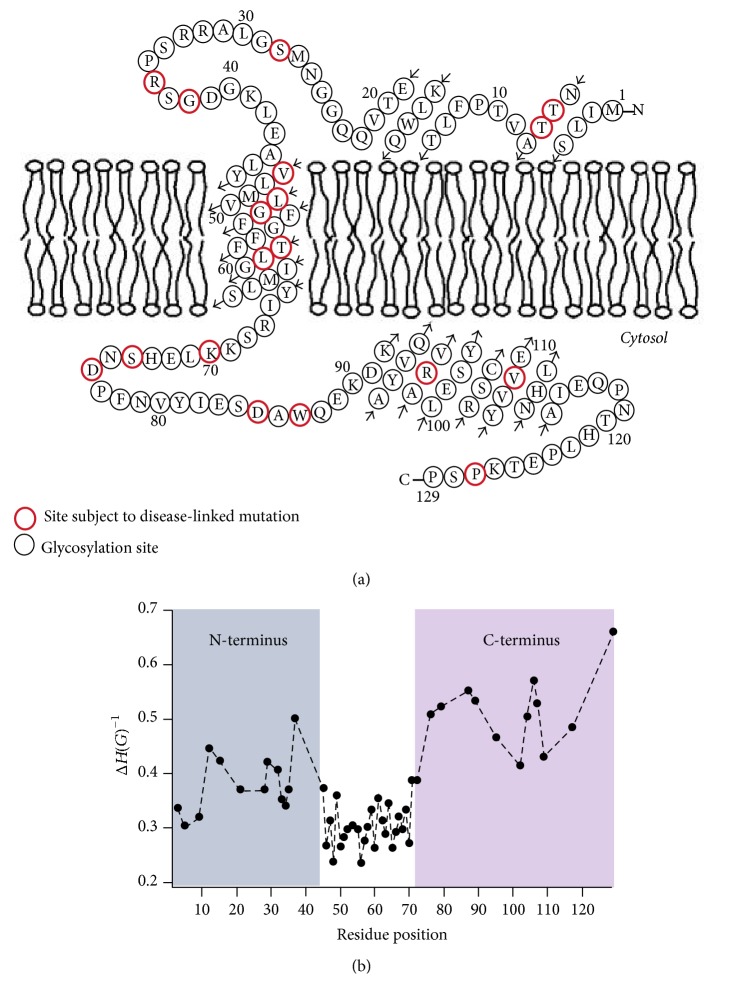
(a) The proposed topology of the KCNE1 sequence in lipid bilayers. (b) Plot of inverse central EPR resonance linewidth (*m*_*I*_ = 0) as a function of residue position of KCNE1 in lipid bilayers (adapted from [51] with permission).

Site-directed spin labeling CW-EPR spectroscopy was recently used to extensively investigate the structural topology and dynamics of KCNE1 in proteoliposomes [51, 68]. KCNE1 is a single pass integral membrane protein which is very important for modulating the functional activities of a voltage gated potassium ion channel (K_v_). It is essential for the cardiac action potential that mediates a heartbeat as well as the potassium ion homeostasis in the inner ear. CW-EPR lineshape analysis was performed on 53 sites of spin labeled KCNE1 including all 27 residues of the transmembrane domain (45–71), and 26 residues of the N- and C-termini of KCNE1 in lipid bilayered vesicles to study the nitroxide side-chain motion. The results indicated that the nitroxide spin label side-chains located in the KCNE1 TMD are less mobile when compared to the extracellular region of KCNE1. The EPR data further revealed that the C-terminus of KCNE1 is more mobile when compared to the N-terminus. EPR power saturation data obtained on 41 sites of spin labeled KCNE1 were used to determine the topology of KCNE1 with respect to the 1-palmitoyl-2-oleoyl-phosphatidylcholine (POPC)/1-palmitoyl-2-oleoyl-phosphatidylglycerol (POPG) lipid bilayers. Also, the data showed that the transmembrane domain is spanning the width of the lipid bilayer, while the extracellular region of KCNE1 is solvent-exposed with some of the portions partially or weakly interacting with the membrane surface.  Figure 4 shows the proposed topology and the spin label side-chain mobility of the KCNE1 sequence in lipid bilayers. The CW-EPR data obtained on KCNE1 in various environments (i.e., 1-myristoyl-2-hydroxy-sn-glycero-3-phospho-(1′-rac-glycerol) (sodium salt) (LMPG) micelles, (POPC)/(POPG) liposomes, and POPC/POPG lipodisq nanoparticles) further suggested that the KCNE1 undergoes multiple conformations while interacting with lipid bilayers [51, 53, 68].

Another recent example of using nitroxide spin labeling CW-EPR spectroscopy is a study on the functional amyloid Obr2A [58]. Obr2A is an isoform of Orb2, having a unique N-terminus domain important for the formation of amyloid-like aggregates and long-term memory in vivo. Soria et al. performed CW-EPR lineshape analysis on several spin labeled sites of Orb2A1-88 in the presence of various lipid concentration to determine the spin label side-chain mobility. The results revealed the increased rigidity of N-terminus of the protein with the increased concentration of lipid vesicles. Their CW-EPR data further revealed that the Orb2A1-88 membrane binding depends on the membrane curvature.

Nitroxide based SDSL CW-EPR spectroscopy at X-band can also be used to study membrane topology of membrane proteins/peptides bound to aligned phospholipid bilayers [69–72]. An excellent example of recent work using this method is the study of phospholamban [72]. McCaffrey et al. applied a bifunctional spin label and X-band EPR spectroscopy on monomeric phospholamban (PLB) to determine the protein structural topology in magnetically aligned bicelles. Phospholamban (PLB) is a single pass integral membrane protein that regulates the cardiac sarcoplasmic reticulum Ca-ATPase (SERCA). The result of this study suggested that the EPR spectra of a bifunctional spin label can be used to accurately determine the orientation and rotational dynamics of an *α*-helical segment of an integral membrane protein in magnetically aligned bicelles. Recently, the Lorigan lab determined accurate helical tilt angle and dynamics of the AchR M2*δ* peptide by utilizing the magnetically aligned bicelles EPR technique and multiple TOAC labeled peptide substitutions [71].

### 2.2. Local Secondary Structure of Membrane Proteins

**Figure 5:**
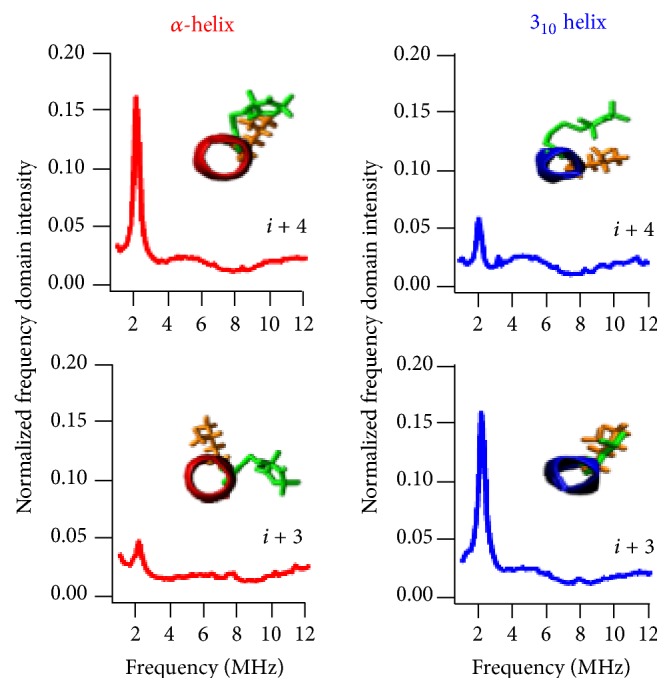
Three-pulse ESEEM FT data of ^2^H-labeled d_10_ Leu4 LRL8 peptide in TFE (*α*-helix) and DPPC liposomes (3_10_-helix) for *i* + 3 and *i* + 4 samples (adapted from [52] with permission).

The assembly, packing, and interaction of membrane proteins with its lipid environment are largely affected by the local secondary structure of membrane proteins. Better information on the local secondary structure is essential for understanding the function, dynamics, and interacting mode of membrane proteins [73, 74]. Nitroxide based SDSL electron spin echo envelope modulation (ESEEM) spectroscopy is a very powerful pulsed EPR spectroscopic technique to measure distances up to 8 Å between a spin label and a single NMR active isotopic nucleus such as deuterium (^2^H) [75, 76]. It has been used to investigate penetration of water into membranes, localization of proteins, or lipids in lipid membranes [77–81]. Recently SDSL ESEEM spectroscopy has been utilized to directly probe the local secondary structure of membrane proteins/peptides in aqueous as well as lipid membrane environments [52, 79, 80, 82–86]. In this method, a cysteine mutated nitroxide spin label is positioned 2 (*i* + 2), 3 (*i* + 3), or 4 (*i* + 4) residues away from a fully deuterated Leu side-chain (*i*). The characteristic periodicity of the *α*-helix (3.6 residue per turn with a pitch of 5.4 Å) and a 3_10_- helix (3.1 residue per turn with a pitch of 6.0 Å) structure gives rise to a unique pattern in the ESEEM spectra. A larger ^2^H ESEEM peak in the FT frequency domain data is observed for the *i* + 4 samples, when compared to the *i* + 3 samples for the *α*-helix, whereas the opposite pattern is revealed for the 3_10_-helix [52]. For all *i* + 2 samples, no ^2^H ESEEM peak in the FT frequency domain data is revealed due to spin labels being too far away from the ^2^H-labeled side-chain to be detected. These unique patterns provide pertinent local secondary structural information to distinguish between the *α*-helical and 3_10_-helical structural motifs for protein/peptides using this ESEEM spectroscopic approach with short data acquisition times (~30 min) and small sample concentrations (~100 *µ*M) as well as providing more site-specific secondary structural information compared to other common biophysical approaches such as CD.  Figure 5 shows an example of the three-pulse ESEEM data obtained for an amphipathic model peptide, LRL_8_ [52]. When LRL_8_ is solubilized in trifluoroethanol (TFE), the peptide adopts an *α*-helical structure and, alternatively, forms a 3_10_-helical secondary structure when incorporated into liposomes. The inset in Figure 5 shows the spatial relationship between the spin label and the ^2^H-labeled Leu residue from MD simulations.

### 2.3. SDSL Distance Measurement of Membrane Proteins

Distance information can be obtained from two spin labels in terms of either intramolecular distances between sites on the same protein or intermolecular distances between sites on different proteins [18]. The distance is obtained from the magnetic dipolar interactions between the unpaired electrons of two spin labels. The energy of the dipolar interaction is inversely proportional to the cube of the distance (*r*^3^). When the distance is less than 20 Å, electron-electron interaction significantly broadens the CW-EPR spectral lineshape. The strength of the interaction is estimated qualitatively from the degree of line broadening using a variety of lineshape analysis techniques and corresponding distance information can be revealed [18, 20, 87–90]. Using dual labeling EPR techniques, distances can be measured to probe secondary, tertiary, and quaternary structures [87]. CW dipolar broadening EPR can provide pertinent structural and functional dynamic information over an intermediate distance range of 8–20 Å [70, 91–93]. Nitroxide based SDSL CW dipolar broadening EPR has been applied to several important biological systems such as bacteriorhodopsin, erythroid *β* spectrin, AchR M2*δ* peptide, magainin 2, bacterial K^+^-translocating protein KtrB,* E. coli* integral membrane sulfurtransferase, and KCNE1 [53, 70, 90, 93–98].

**Figure 6:**
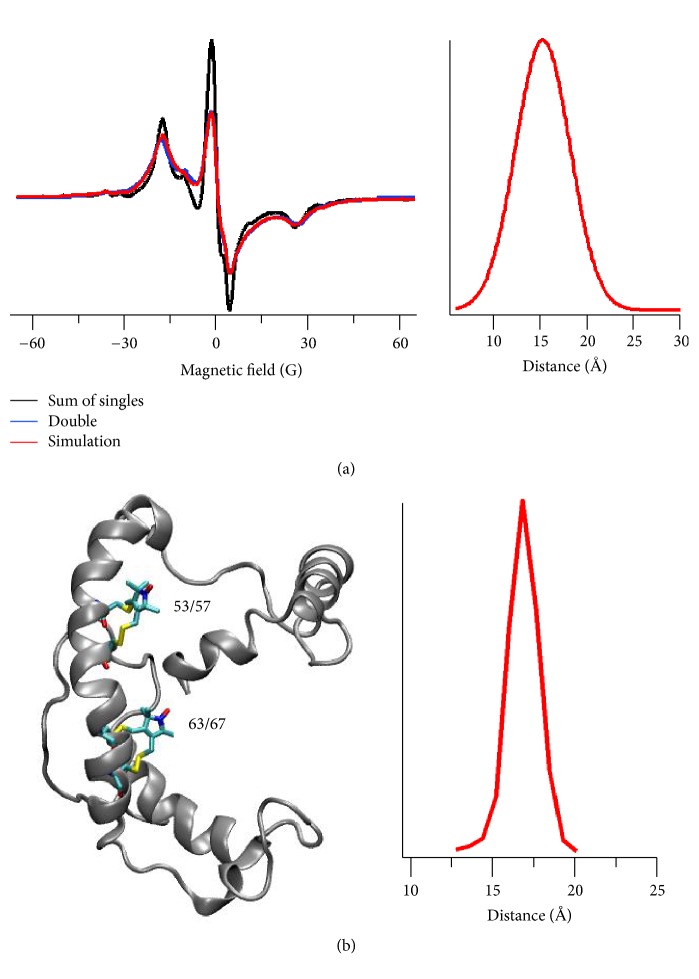
CW dipolar broadening EPR study of KCNE1. (a) CW dipolar broadening EPR spectra of KCNE1 bearing BSLs at sites 53/57 and 63/67 in POPC/POPG liposomes (left panel) and the corresponding distance distribution (right panel) obtained from data analysis by using the Short Distances LabVIEW program. (b) Cartoon representation of KCNE1 bearing two BSLs at sites 53/57 and 63/67 (left panel) and the corresponding distance distribution obtained from 20 ns molecular dynamics trajectory data analysis (right panel) (adapted from [53] with permission).

A recent example of using SDSL CW dipolar broadening EPR is the study of an integral membrane protein KCNE1 [53]. Sahu et al. applied CW dipolar broadening EPR technique to measure a distance between two bifunctional spin label (BSLs) attached on the transmembrane domain of KCNE1 in lipid bilayers at room temperature. The experimental result was further validated using a 20 ns molecular dynamics modeling study.  Figure 6 shows CW dipolar broadening EPR data on KCNE1 in POPC/POPG lipid bilayers at room temperature. The CW dipolar line broadening EPR data revealed 15 ± 2 Å distance between doubly attached BSLs on KCNE1 (53/57-63/67) which is consistent with molecular dynamics modeling and the solution NMR structure of KCNE1 which yielded a distance of 17 Å. This study demonstrated the utility of investigating the structural and dynamic properties of membrane proteins in physiologically relevant membrane mimetics using BSLs.

For longer distances, pulsed EPR double electron-electron resonance (DEER) spectroscopy has been a widely used biophysical technique for measuring distances between 20 and 80 Å [99, 100]. Distance measurements are one of the most popular and rapidly expanding aspects of SDSL EPR spectroscopy. DEER is also known as pulse electron double resonance (PELDOR). In DEER, the measurement of the coupling between the two spins can be performed by monitoring one set of spins while exciting another set of spins with a second microwave frequency that leads to the measurement of distance between them. SDSL DEER spectroscopy has been applied for probing the structure and conformational dynamics of a wide variety of biological systems [9, 99–107]. It can also be used to measure the relative orientation of the spins when the experiment is performed at higher magnetic field of 94 GHz [108]. Although the DEER technique is very popular in the structure biology field, the technical limitations of membrane protein sample preparation in its functional environment introduce challenges in accurate and precise distance measurements. The challenges arise due to much shorter transverse relaxation/phase memory times due to the heterogeneous distribution of spin labeled proteins within the membrane creating local inhomogeneous pockets of high spin concentration and poor DEER modulation in more biologically relevant liposomes when compared to water soluble proteins or membrane proteins in detergent micelles [104]. Additionally, the use of a high effective protein concentration in the liposome samples introduces a strong background contribution causing extreme limits on sensitivity, distance range, and experimental throughput [109]. The protein backbone dynamics and spin label rotameric motions have also a significant contribution to the DEER distance distribution width.

In recent years, impressive studies have been done to overcome these challenges using membrane protein reconstitutions in the presence of unlabeled proteins, bicelles, nanodiscs, lipodisq nanoparticles, a low protein/lipid molar ratio, restricted spin label probes, and Q-band pulse EPR measurements [9, 106, 110–116]. Recently, several labs have utilized DEER distance restraints coupled with molecular dynamics simulations to refine the structure of membrane proteins [56, 106, 117]. These methodological developments have made DEER a powerful and popular structure biology tool to study complicated biological systems such as membrane proteins. SDSL DEER spectroscopy has been applied to study several important membrane protein systems such as bacteriorhodopsin, KCNE1, KCNE3, C99 Amyloid Precursor Protein, KvAP voltage-sensing domain, human dihydroorotate dehydrogenase enzyme (HsDHODH), Influenza A M2 protein, cardiac Na^+^/Ca^2+^ exchange (NCX1.1) protein, Na^+^/Proline Transporter PutP* Escherichia coli*, tetrameric potassium ion channel KcsA, *α*-Synuclein, and ABC Transporter MsbA [56, 106, 110–112, 117–124]. SDSL DEER spectroscopy has been recently used to study the oligomerization states of several membrane proteins such as NhaA Na+/H+ antiporter of* E. coli*, KcsA, M2 transmembrane domain, LptA, and proteorhodopsin [60, 103, 119, 125, 126]. This is a very powerful biophysical technique to determine the oligomeric structure of membrane proteins.

A recent application of SDSL DEER spectroscopy is the conformational study of KCNE3 in proteoliposomes [122]. KCNE3 is a single pass transmembrane protein that modulates a variety of voltage gated ion channels in diverse biological contexts. In epithelial cells, KCNE3 regulates the function of the KCNQ1 potassium ion (K^+^) channel in a physiologically critical cellular transport process in several organs and whose malfunction causes diseases such as cystic fibrosis (CF), cholera, and pulmonary edema. Kroncke et al. performed SDSL DEER experiments on the protein in different membrane environments to identify a curved *α*-helical nature of the transmembrane of KCNE3 [122]. Two MTSL spin labels were generated at the ends of the transmembrane domain (TMD) of KCNE3. The dual spin labeled KCNE3 protein was reconstituted into micelles [lyso-myristoylphosphatidylcholine (LMPC)], bicelles (DMPG/DHPC), and 1-palmitoyl-2-oleoyl-phosphatidylcholine (POPC)/1-palmitoyl-2-oleoyl-phosphatidylglycerol (POPG) lipid bilayers. The resulting distances for all three media were statistically identical. These results indicated that the curvature of the TMD is an intrinsic property of KCNE3 that is maintained in lipid bilayers, bicelles, and detergent micelles. The TMD curvature of KCNE3 is important for the kinetics of initial binding of KCNE3 to the KCNQ1 channel.

Another excellent recent application of SDSL DEER spectroscopy is the study of conformational dynamics of a multidrug transporter LmrP from* Lactococcus lactis* [127]. LmrP is a member of the major facilitator superfamily (MFS). It couples the downhill translocation of protons along their transmembrane gradient to the uphill transport of hydrophobic cytotoxic compounds. The active efflux of diverse cytotoxic compounds through multidrug transporters contributes to bacterial antibiotic resistance. Martens et al. performed DEER distance measurements on the selected spin label pairs of the transporter reconstituted into nanodiscs of different lipid compositions [127]. The resulting DEER data revealed the conformational energy landscape of the transporter modulated by the lipid headgroups. The results further suggested a direct interaction between lipid headgroups and a conserved motif of charged residues that control the conformational equilibrium through an interplay of electrostatic interactions within the protein. Although SDSL EPR has several advantages over the existing biophysical techniques for studying membrane proteins, it needs an incorporation of spin label probes which might not be suitable for all the desired sites on some membrane protein systems due to low spin labeling efficiency.

## 3. Conclusion

In this review, we discussed some recent applications of nitroxide based SDSL EPR spectroscopic techniques to study important membrane protein systems. SDSL EPR spectroscopy is very popular and growing structure biology technique used to answer pertinent structural and dynamic questions related to biological systems. It can provide important information on complicated biological systems which is very challenging or nearly impossible by using other biophysical techniques.
